# *NAPRT* Expression Regulation Mechanisms: Novel Functions Predicted by a Bioinformatics Approach

**DOI:** 10.3390/genes12122022

**Published:** 2021-12-20

**Authors:** Sara Duarte-Pereira, Olga Fajarda, Sérgio Matos, José Luís Oliveira, Raquel Monteiro Silva

**Affiliations:** 1 Department of Medical Sciences, iBiMED—Institute of Biomedicine, University of Aveiro, 3810-193 Aveiro, Portugal; 2 IEETA—Institute of Electronics and Informatics Engineering of Aveiro, University of Aveiro, 3810-193 Aveiro, Portugal; olga.oliveira@ua.pt (O.F.); aleixomatos@ua.pt (S.M.); jlo@ua.pt (J.L.O.); 3 DETI—Department of Electronics, Telecommunications and Informatics, University of Aveiro, 3810-193 Aveiro, Portugal; 4 Universidade Católica Portuguesa, Faculty of Dental Medicine, Center for Interdisciplinary Research in Health (CIIS), 3504-505 Viseu, Portugal; rmsilva@ucp.pt

**Keywords:** NAPRT (nicotinate phosphoribosyltransferase), bioinformatics, expression regulation, cell differentiation, neurodevelopment

## Abstract

The nicotinate phosphoribosyltransferase (*NAPRT*) gene has gained relevance in the research of cancer therapeutic strategies due to its main role as a NAD biosynthetic enzyme. NAD metabolism is an attractive target for the development of anti-cancer therapies, given the high energy requirements of proliferating cancer cells and NAD-dependent signaling. A few studies have shown that *NAPRT* expression varies in different cancer types, making it imperative to assess *NAPRT* expression and functionality status prior to the application of therapeutic strategies targeting NAD. In addition, the recent finding of NAPRT extracellular form (eNAPRT) suggested the involvement of NAPRT in inflammation and signaling. However, the mechanisms regulating *NAPRT* gene expression have never been thoroughly addressed. In this study, we searched for *NAPRT* gene expression regulatory mechanisms in transcription factors (TFs), RNA binding proteins (RBPs) and microRNA (miRNAs) databases. We identified several potential regulators of *NAPRT* transcription activation, downregulation and alternative splicing and performed GO and expression analyses. The results of the functional analysis of TFs, RBPs and miRNAs suggest new, unexpected functions for the *NAPRT* gene in cell differentiation, development and neuronal biology.

## 1. Introduction

Nicotinate phosphoribosyltransferase (NAPRT) is an enzyme from NAD (Nicotinamide Adenine Dinucleotide) biosynthesis and is mostly studied as a cancer biomarker.

One of the cancer therapeutic strategies targeting NAD metabolism is the use of nicotinamide phosphoribosyltransferase (NAMPT) inhibitors [1,2,3,4]. NAMPT is the rate-limiting enzyme of the main NAD salvage pathway, which uses nicotinamide, while NAPRT is responsible for NAD production via the nicotinic acid precursor, known as the Preiss–Handler pathway [5,6]. In this context, NAPRT became an important biomarker for the use of nicotinic acid as a co-adjuvant in NAPRT-negative tumors [1] and for co-inhibition in the cancer types that highly express NAPRT [7].

Lack of *NAPRT* expression was observed in several cancer types [1,8,9,10] and associated with *NAPRT* epigenetic silencing in some cases, such as gastric and lung cancer [10,11,12,13]. On the other hand, *NAPRT* amplifications and overexpression were reported in ovarian, breast and pancreatic cancer [7]. Differences in *NAPRT* expression between subtypes of cancer, namely in breast, pancreatic, lung and gastric carcinomas [7,11,14,15], suggest that individual variability should be considered in therapeutic approaches. In order to select the types of cancer that would better respond to NAMPT inhibition, a determination should be made on whether NAPRT is expressed and functional. 

In addition to cancer, the role of NAPRT in inflammation and signaling was recently discovered and is brought by its extracellular form (eNAPRT) [16,17]. Thus, knowledge of *NAPRT* gene expression regulatory mechanisms under non-pathological conditions is key to understanding its biological functions and roles in disease, in addition to exploring its potential as a biomarker or therapeutic target. 

We have previously studied *NAPRT* methylation and mutations in cancer [10,18]. In addition to promotor methylation, mutations in transcription factor binding sites (TFBS) and alternative splicing have been proposed as potential regulatory mechanisms of *NAPRT* gene expression [10,11,14,18]. As *NAPRT* gene expression variation is tissue-specific rather than a cancer specific alteration [15], we searched several databases of transcription factors (TFs), RNA binding proteins (RBPs) and microRNAs (miRNAs), using the *NAPRT* gene as a target to identify additional transcriptional and post-transcriptional mechanisms in normal conditions. We identified several potential regulators of *NAPRT* transcription activation, downregulation and alternative splicing. The analyses of their targets suggested unexpected functions in cell differentiation, development and neuronal biology. 

## 2. Methods

### 2.1. Collection of NAPRT Potential Regulators

In order to obtain a comprehensive set of results, we searched a diverse number of databases in our approach, which are often focused on different methodologies: some collect data from experimental high throughput studies, others used computational algorithms to predict binding and others extracted data obtained directly from the literature. The databases used here are summarized in Supplementary Table S1.

#### 2.1.1. Transcription Factors

In order to search for TFs that could regulate *NAPRT* gene expression, we surveyed one database with experimental data from the Encyclopedia of DNA Elements (ENCODE) Project [19]—Factorbook [20]—and four sources of computational predictions of transcription factor binding sites (TFBS)—UniPROBE [21]—PROMO v3.0.2, which uses the TFBS defined in the TRANSFAC database v8.3 [22,23], MotifMap [24,25] and CTCFBS v2.0 [26], using *NAPRT* 5′UTR sequence as an input. All unique results were considered.

#### 2.1.2. RNA Binding Proteins

We collected data from a total of eight databases. Four of them included experimentally validated data—starBase v2.0, currently called The Encyclopedia of RNA Interactomes (ENCORI) [27], miRWalk v2.0 [28], CLIPdb v1.0 [29], currently integrated in POSTAR2 [30] and AURA v2.4 [31]. The other four had computationally predicted data—RBPDB v1.3 [32], RBPmap v1.0 [33], catRAPID [34] and CISBP-RNA v0.6 [35]. Starbase and miRWalk also provided information on microRNA–mRNA interactions.

In most of the queries, we searched for the entire *NAPRT* mRNA sequence and then performed a specific search using only 3′UTR or 5′UTR sequences due to their regulatory role. For some databases, such as miRWalk, the query for the gene symbol *NAPRT* or *NAPRT1* did not retrieve any results; thus, other identifiers were used, such as EntrezID, Ensembl gene ID or UniProt accession.

#### 2.1.3. microRNAs

We searched for experimentally validated data in DIANA-TarBasev7.0 [36], starBase v2.0 [27] and mirTarBase 6.0 [37]. For computationally predicted miRNA:mRNA interactions, we used DIANA-microT-CDS v5.0 [38] and TargetScan 7.0 [39]. From miRWalk v2.0 database [28], we retrieved both experimentally validated and predicted data. The best targets from the computational predictions were selected according to the following criteria: the binding site should be 8 nucleotides, and the conservation of the site sequence among different species should be the highest (in the case of TargetScan a conserved branch length over 0.8 for 8 nucleotides is recommended).

In addition, we obtained expression data and performed further analysis using DASHR v2.0 [40], starBase v2.0 [27], miRTarBase 6.0 [37] and miRGator v3.0 [41].

### 2.2. Data Analysis

**Figure 1:**
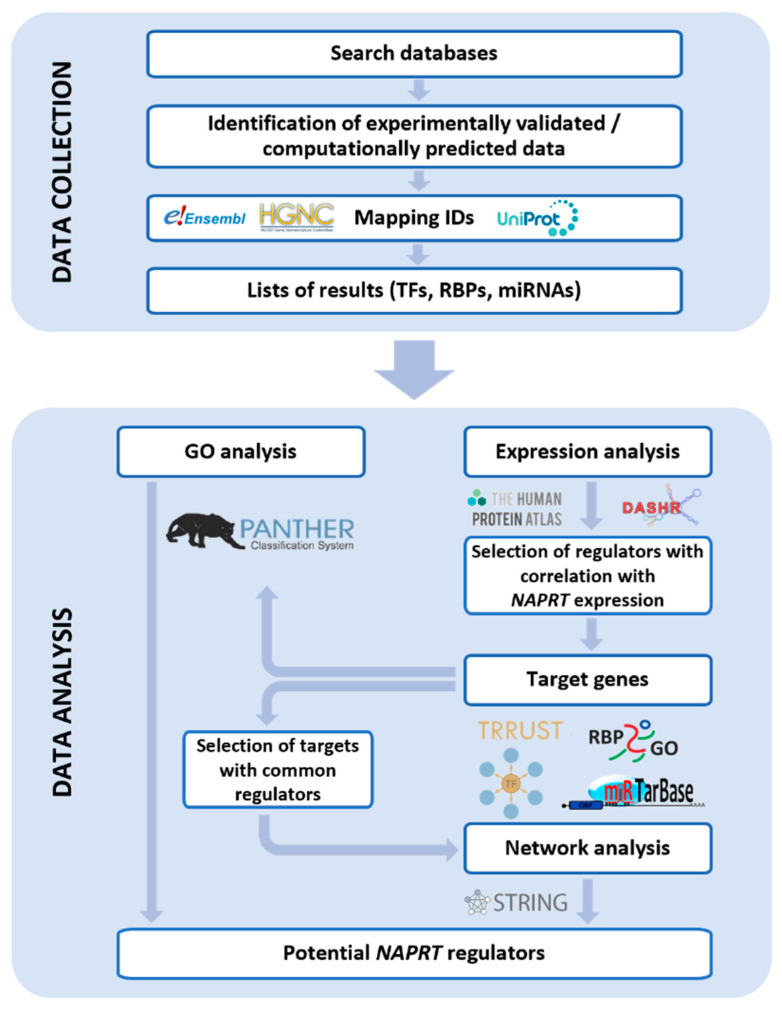
Data analysis. Schematic representation showing the pipeline followed to collect and analyze nicotinate phosphoribosyltransferase (*NAPRT*) putative binding transcription factors (TFs), RNA binding proteins (RBPs) and microRNAs (miRNAs). The list of databases can be found in Supplementary Table S1.

After collecting data from the various databases (Supplementary Table S1), the results were further studied as follows (Figure 1).

For TFs and RBPs, we used the HUGO Gene Nomenclature Committee (HGNC) at the European Bioinformatics Institute (www.genenames.org, accessed on 29 April 2021) and the UniProt (www.uniprot.org, accessed on 12 July 2021 [42]) databases to map each gene symbol to HUGO, Ensembl and UniProt IDs and retrieved information of the subcellular location, evidence level of expression, tissue specificity and function. 

The study of gene co-expression patterns, the analysis of co-regulation networks and gene ontology enrichment analysis are commonly used methodologies to infer knowledge on gene functions. Thus, we analyzed the correlation between *NAPRT* gene expression and the expression of the TFs, RBPs and miRNAs from our set of results and expanded our study to the analysis of the target genes of those regulators, as described below.

#### 2.2.1. Correlation with NAPRT Gene Expression 

Expression data were retrieved from the Human Protein Atlas (www.proteinatlas.org, accessed on 24 January 2021, v19.3 [43]). The dataset containing the consensus normalized expression levels summarize expression values for 62 non-pathological tissues based on transcriptomics data from three sources (Human Protein Atlas, Gene Tissue Expression (GTEx) and FANTOM5), and it was used to calculate the correlation between the expression of *NAPRT* gene and the identified *NAPRT* TFs and RBPs. In order not to assume linear relationships between co-expressed genes, Spearman’s correlation was calculated. Positive correlation values above 0.5 and negative correlation values below −0.5 were considered [44]. For miRNAs, we assessed expression data from DASHR v2.0 [40] and obtained the analysis of miRNA correlation with *NAPRT* expression using miRGator v3.0 [41]. 

#### 2.2.2. Target Genes

For the TFs and RBPs that presented a significant correlation with *NAPRT* expression, we used the TRRUST v.2 database (grnpedia.org/trrust/, accessed on 9 March 2021 [45]) and the RBP2GO database (rbp2go.dkfz.de/, accessed on 26 March 2021 [46]) to search for their target genes. The gene set enrichment analysis of the target genes of the experimentally validated miRNAs was performed with miRTarBase 6.0. 

#### 2.2.3. Functional Analysis

We performed a Gene Ontology (GO) analysis on the total lists of TFs, RBPs and the target genes of the expression-correlated TFs, RBPs and miRNAs using a statistical overrepresentation test (Fisher’s exact test and False Discovery Rate correction) in the PANTHER Classification System (pantherdb.org, accessed on 4 October 2021, v.16.0 [47]). In the case of the TFs, we created a combined dataset to use as background in GO analysis. For that purpose, we used three sources, namely, the Human Protein Atlas [43] annotated transcription dataset (proteinatlas.org/search/protein_class%3Atranscription+factors, accessed on 4 January 2021); the list of TFs from the ChIP-seq Peaks from the ENCODE Project [48] (encodeproject.org/, accessed on 4 January 2021, source data version: ENCODE 3 November 2018); and the human transcription factors catalogue from Lambert et al. 2018 [49]. In the case of the RBPs, we used the dataset published on the RBP2GO database [46]. On the remaining analysis, all genes in the genome were used as the enrichment background. We preferably used the PANTHER slim annotation datasets for biological processes and molecular functions, but whenever there were no statistically significant results (FDR corrected *p* value < 0.05), we chose complete annotation datasets [47].

The target genes that were common to more than one mechanism, e.g., that were targeted by at least one TF and one RBP, one TF and one miRNA or one RBP and one miRNA, were analyzed on a network of interactions obtained by using STRING (string-db.org, accessed on 8 December 2021, v.11 [50]). We searched for data from text-mining, experiments, databases and co-expression, with a high confidence level of 0.7. Only interactions between the queried proteins were considered.

## 3. Results

### 3.1. Potential Regulators of NAPRT Gene Expression

#### 3.1.1. Transcription Factors

From a total of 93 results obtained from the four databases surveyed, 80 were mapped to UniProt IDs (Supplementary Table S2). Thirty-seven of them had supporting experimental data, which derived from the ENCODE project (Supplementary Table S3). Three were common to two databases: Wilms tumor protein (WT1), Erythroid transcription factor GATA-binding factor 1 (GATA1) and YY1 transcription factor (YY1), also known as Yin and Yang 1 protein. 

In order to exclude potential overrepresentation of the typical functions and biological processes in which TFs are usually involved, we used a large dataset of human TFs as background in the GO analysis. This reference dataset was compiled from three sources and resulted in 1849 known human TFs. In addition to the expected processes related to the transcription regulation and the involvement in the RNA metabolic process, GO analysis of the 80 TFs revealed an overall overrepresentation for genes involved in signaling and response to stimulus (Supplementary Table S4), such as “response to UV-C” or “positive regulation of defense response to virus by host”. Table 1 lists the most relevant biological processes enriched (considering a fold enrichment of over 3 with 10 or more genes). 

**Figure 2:**
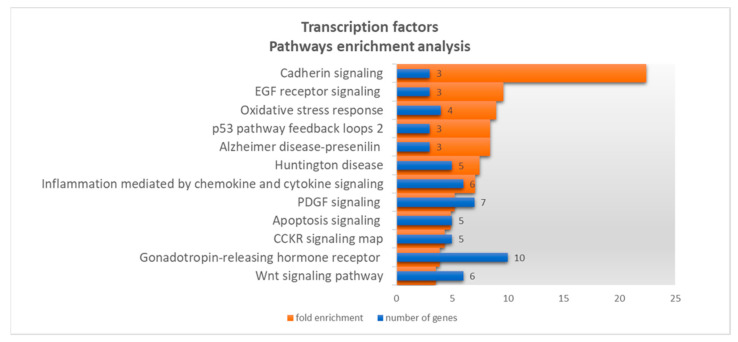
Gene ontology results for 80 potential *NAPRT* transcription factors, based on pathways annotation dataset, showing the number of genes (blue bars) and fold enrichment (orange bars) by decreasing fold enrichment order. Detailed information on GO results can be found in Supplementary Table S4. The genes responsible for the highest enrichment fold were retrieved (TCF7L2, TCF3 and LEF1).

Considering pathway annotations (Figure 2), the highest enrichment fold was provided by three genes involved in the cadherin signaling pathway, namely, Transcription factor 7-like 2 (TCF7L2), Lymphoid enhancer binding factor (LEF1) and Transcription factor 3 (TCF3). Curiously, the set of the same three genes was responsible also for the enrichment of the Alzheimer disease presenilin pathway and the Wnt signaling pathway. In the latter, we found three additional genes: Tumor protein p53 (TP53), MYC proto-oncogene and Nuclear factor of activated T cells 1 (NFATC1). Of note, from these TFs, TCF7L2, MYC and NFATC1 have been experimentally validated.

#### 3.1.2. RNA Binding Proteins

From a total of 122 RBPs (Supplementary Table S5), we obtained 113 RBPs predicted to bind *NAPRT* mRNA and 11 with experimentally supported data (Supplementary Table S6). Only splicing factor U2AF 65 kDa subunit (U2AF2) and the FUS RNA binding protein (FUS) were both predicted and validated. Three RBPs, namely Heterogeneous nuclear ribonucleoprotein L (HNRNPL), Protein quaking (QKI) and RNA-binding motif protein 3 (RBM3), were predicted to bind specifically to *NAPRT* 3′UTR; that is, no binding site was found in the remaining mRNA sequence. 

We retrieved information on subcellular location, evidence level of expression, tissue specificity and function for each of the 11 experimentally validated RBPs. Several of these are reported to be functionally involved in post-transcriptional processing, as it was expected, and have ubiquitous expression. Some are necessary for normal splicing events, such as Eukaryotic initiation factor 4A-III (EIF4A3) and U2AF2, and Serine/arginine repetitive matrix protein 4 (SRRM4) is a splicing factor specifically required for neural cell differentiation. 

**Figure 3:**
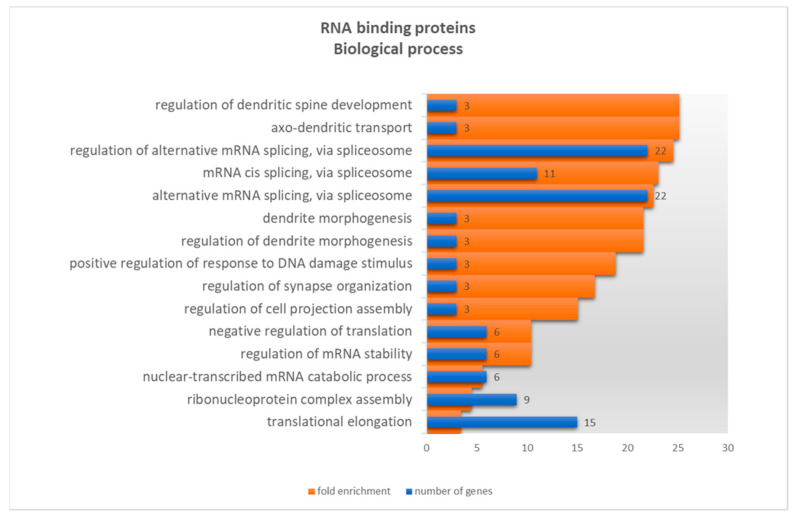
Gene ontology results for 122 potential *NAPRT* RNA binding proteins, based on biological processes annotation dataset, showing the number of genes (blue bars) and fold enrichment (orange bars) by decreasing fold enrichment order. Only child terms are presented. Detailed information on GO results can be found in Supplementary Table S7. The genes responsible for the highest enrichment fold were retrieved (FXR1, FXR2 and FMR1).

Despite the use of a reference dataset composed by all potential human RBPs, GO analysis of the 122 *NAPRT*-binding RBPs still showed several processes related to splicing mechanisms, regulation of alternative splicing, splice site selection, regulation of translation and regulation of mRNA stability (Figure 3 and Supplementary Table S7). These results reflect the well-known functions of RBPs and the previously described alternative splicing events that regulate *NAPRT* expression [10]. Unexpectedly, several processes related to dendrite development and synapse organization were also found. The highest fold enrichment was retrieved for term regulation of dendritic spine development and axo-dendritic transport, associated with the functions of Fragile X mental retardation syndrome-related proteins 1 (FXR1) and 2 (FXR2) and Synaptic functional regulator (FMR1). Other processes related to dendrite morphogenesis and synapse organization were also enriched.

#### 3.1.3. microRNAs

A total of 39 miRNAs that potentially bind *NAPRT* were obtained in this study (Supplementary Table S8), from which seven have experimental data (Supplementary Table S9). Two of them were obtained from two different databases (miR-218-5p and miR-92a-3p). From the 31 miRNAs based on computational predictions, miR-491-5p was the only prediction that followed the established criteria and was selected for further analysis, along with the seven miRNAs based on experimental data.

For eight selected miRNAs, DASHR database was used to extract data on miRNAs expression in different tissues, which can be visualized as heatmaps (Supplementary Figure S1). The results showed that most miRNAs appear to be tissue specific. For instance, the brain tissue presents the highest values for miR-197-3p, miR-218-5p, miR-491-5p and miR-92-3p (Supplementary Figure S1a–d). This is most relevant in the case of miR-491-5p (Supplementary Figure S1d), which has an overall low expression, except for brain. Of note, miR-218-5p is weakly expressed in tissues where *NAPRT* is strongly expressed, such as liver and blood (Supplementary Figure S1c).

### 3.2. Expression Correlation between TFs, RBPs, miRNAs and the NAPRT Gene

Spearman correlation analysis identified 11 TFs and 9 RBPs with a significant positive correlation, from moderate to strong, with *NAPRT* expression, based on the Human Protein Atlas dataset of non-pathological human tissues (Table 2). With the exception of the zinc finger and BTB domain containing 7A (ZBTB7A), which acts as a repressor, all these TFs can both activate or repress their target genes. Most of the RBPs are involved in splicing or in the general process of transcription, and many of them are a part of the hnRNP family. The zinc finger CCHC-type containing 17 (ZCCHC17) was the only RBP that presented a significant negative correlation with *NAPRT* expression.

We also found a significant correlation between miR-92a-3p and miR-218-5p and *NAPRT* expression in different datasets (Table 3). Interestingly, miR-92a-3p had a positive or a negative correlation depending on tissues. The strongest correlation was positive and was found in the dataset of differentiated embryonic stem cells.

### 3.3. Analysis of Target Genes

Next, we identified 340 known targets for the 11 TFs that correlate with NAPRT expression (Supplementary Table S10). There were no reported targets only for the transmembrane protein 37 (TMEM37). Forty-three target genes were regulated by two or more TFs. For the nine RBPs positively correlated with *NAPRT* expression, we obtained 148 target genes, and one more for the negatively correlated RBP (Supplementary Table S11). Fifty-five of them were targeted by two or more RBPs. Among them, we found many heterogeneous nuclear ribonucleoproteins (hnRNPs), small nuclear ribonucleoproteins (snRNPs) and serine/arginine-rich splicing factors (SRSFs).

In miRTarBase, we performed Gene Set Enrichment analysis for all target genes of a given miRNA. For each of the eight miRNAs studied, we selected the genes with at least two validation methods, resulting in 1 gene for miR-1915-3p, 63 genes for miR-197-3p, 37 genes for miR-218-5p, 8 genes for miR-491-5p and 51 genes for miR-92a-3p. Between them, only the TP53 gene was targeted by miR-92 and miR-491. The total list of 158 miRNA targets is found on Supplementary Table S12).

Excluding duplicates, we obtained a total of 626 target genes.

#### 3.3.1. Gene Ontology

We performed GO on the collection of all target genes, including the targets of the 11 TFs, the targets of the nine RBPs positively correlated with NAPRT expression and the RBP negatively correlated with NAPRT expression and the targets of the four miRNAs for which we found validated results in a total of 626 genes. Table 4 lists the most relevant biological processes enriched (fold enrichment of over 3 with 10 or more genes), and the complete results are found in Supplementary Table S13. The highest number of genes, 32, was mapped to the inflammation mediated by chemokine and cytokine signaling pathway, but several signaling pathways appeared, including cholecystokinin receptor (CCKR), gonadotropin-releasing hormone receptor (GnRHR), Toll receptor and interleukin pathways. A few key cellular pathways related to both proliferation and apoptosis were also enriched, such as p53, Ras and TGF-β signaling. In addition, we found Alzheimer’s disease and Huntington disease pathways, blood coagulation and angiogenesis within the most relevant results (considering a fold enrichment of above 3, with 10 or more genes).

For miRNAs, a separate GO analysis of each set of targets provided significant results only for miR-218 and miR-92. The terms with the highest fold enrichment were all related to differentiation or development. The terms with the highest number of genes were not only mostly related to the regulation of transcription but also proliferation and differentiation terms (Supplementary Table S14). Overall, GO analysis revealed an overrepresentation for genes involved in developmental processes, mostly of cardiovascular and nervous systems. Genes related to cell differentiation and proliferation, mainly of nervous system and epithelium, were also enriched. In addition, we found several processes related to signaling, including the Wnt pathway, cell adhesion and cell death.

#### 3.3.2. Targets Co-Regulated

We identified 20 genes that were targeted by at least two different mechanisms within our set of results, e.g., a pair of TF/RBP, TF/miRNA or RBP/miRNA. The unique gene found within the target genes of the three types of regulators was cadherin 1 (CDH1).

**Figure 4:**
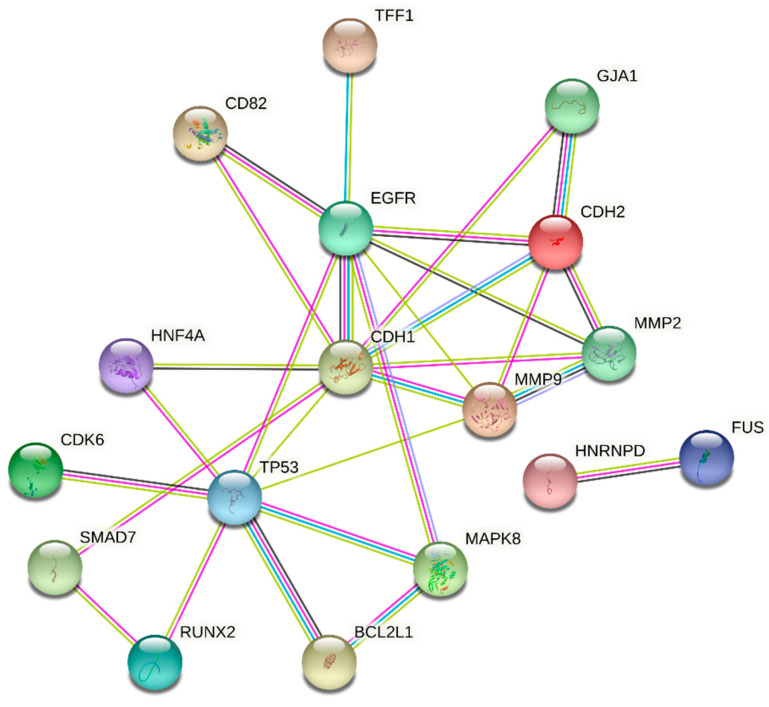
Network of interactions between 17 genes targeted by more than one *NAPRT* potential regulator. The network was obtained by using STRING (string-db.org). The line color of the edges indicates the type of interaction evidence: experimentally determined (pink), from curated databases (light blue), text mining (light green), co-expression (dark grey) and protein homology (purple). The color of the nodes is arbitrary.

In order to find potential associations between these 20 target genes, we used the STRING platform to obtain an interaction network (Figure 4). For 3 out of the 20 genes, no interactions were found (ABCC3, IL1R1 and ZNF175). We retrieved 30 interactions (edges) between 17 genes (nodes), with an average degree of 3 and a significant protein-protein interaction enrichment *p*-value (1.45 × 10^−12^). Eleven out of the twenty proteins were found to have two or more interactions, TP53 being the one with the highest number of interactions (nine).

## 4. Discussion

The goal of this study was to investigate the mechanisms responsible for the regulation of *NAPRT* expression. A thorough survey resulted in identifying a group of TFs, RBPs and miRNAs that potentially bind to the *NAPRT* gene or mRNA, regulating its expression at transcriptional and post-transcriptional levels. For an interaction to occur, the target mRNA and the protein (in the case of TFs and RBPs) or the miRNA must be expressed in the same tissue, which resulted in additional gene expression and network analyses. Based on the rationale of co-regulatory networks of gene expression [51], co-regulated genes are genes that are regulated by at least one common mechanism and are likely to participate in similar biological functions. Tissue expression specificity can also provide important insights into function. Herein, we directed the study to a normal, physiological and non-pathological context. Surprisingly, functional analysis of *NAPRT* regulators uncovered a link with developmental processes, cell differentiation and cell proliferation, which are common to the three mechanisms (TFs, RBPs and miRNAs). In addition, we found that the Cadherin signaling, which was previously associated to *NAPRT* [14], also correlated to *NAPRT* expression regulators.

Among our results, we found several general regulators, such as MYC and TP53, which are two ubiquitous TFs that are known to activate the transcription of growth-related genes and are involved in cell proliferation/apoptotic regulation processes. In the set of RBPs, we could find numerous heterogeneous nuclear ribonucleoproteins (hnRNPs), serine/arginine (SR) proteins and RNA binding motif (RBM) proteins, which are major families of splicing factors. *NAPRT* gene has a high number of alternatively spliced transcripts, which are probably tissue specific, mostly in the brain [10]. This could explain the high number of RBPs involved in splicing that were found to potentially bind *NAPRT*.

Additionally, the GO analysis of TFs showed an enrichment in cadherin signaling and in immune response, as several processes relate to viral response and interleukin production, and the GO analysis of the RBPs suggest an association to dendrite development and synapse organization. In more detail, the most relevant genes found in these analyses were the TFs TCF7L2, TCF3 and LEF1; and RBPs FXR1, FXR2 and FMR1, as these were responsible for the enrichment of the processes with the highest fold. TCF3 is involved in lymphocyte development, and TCF7L2 has been implicated in neural development and diseases [52], such as multiple sclerosis, where a role in demyelination and remyelination has been suggested [53]. LEF1 is specifically expressed in lymphoid tissues, and it activates transcription in the presence of β-catenin (CTNNB1). They participate in the Wnt signaling pathway, which is further discussed below. FXR1, FXR2 and FMR1 belong to the Fragile X family of RBPs. Their role in neurogenesis has been reviewed elsewhere [54]. In addition to Fragile X Syndrome, they have been associated with other neurological disorders and cancer [55].

Correlation analysis with *NAPRT* gene expression revealed eleven TFs and ten RBPs, which would have a greater probability of exerting a regulatory mechanism in *NAPRT* transcription activation and processing (Table 2). RBP ZCCHC17, which has unique negative correlation with *NAPRT* expression, has been scarcely studied, but the most relevant studies place it as a regulator in Alzheimer’s disease [56]. Of note, signaling pathways related to Alzheimer’s and Huntington’s diseases, both degenerative brain diseases, also appeared in the GO analysis.

Among the positively *NAPRT* correlated factors, only TFs BCL3, PML and YY1 were identified in experimentally validated data. *BCL3* gene encodes for a transcription co-activator, previously known as B-Cell Lymphoma 3-Encoded Protein, due to its role in lymphoma and leukemia. It was initially implicated in cell lineage determination [57] and more recently associated with Wnt/β-catenin signaling in other types of cancer [58]. A positively correlated RBP was the epithelial splicing regulatory protein 2 (ESRP2), which particularly regulates the splicing of transcripts that undergo changes in splicing during epithelial-to-mesenchymal transition (EMT), namely CD44 and CTNND1. Among the TFs correlated with *NAPRT* expression, ZBTB7A (Zinc Finger and BTB Domain Containing 7A) was the only TF that acts only as a repressor (and not activator), according to the TRRUST database. It represses SLC2A3 (GLUT3), a glucose membrane transporter, which is mostly known for its specific expression in neurons. Very recently, GLUT3’s role in glioblastoma has been described [59].

GO analysis results of the target genes of the miRNAs miR-218-5p and miR-92a-3p are consistent with the results for TFs and RBPs, where an enrichment in neural and cardiac development and cell differentiation was found, and a considerable number of processes related to signaling included the Wnt pathway.

Although most research studies on these microRNAs are related to cancer, where they have been identified as tumor suppressors, other areas are being investigated. MiR-218 was described as motor-neuron specific and its downregulation was associated with neurodegeneration in mice [60] and amyotrophic lateral sclerosis in humans [61]. In association with the Wnt/b-catenin pathway, miR-218 is involved in osteogenic differentiation [62], in neuronal differentiation of adipose stem cells [63] and, more recently, it was shown that miR-218 is required for functional neural-like cells [64]. MiR-92 is also involved in processes of EMT, particularly related to angiogenesis [65]. Moreover, miR-491 is associated with the post-transcriptional regulation of dopamine transporters in neural cells [66]. In addition, miR-491 is among the miRNAs identified as regulators of the transcription factor TCF7, which acts in the Wnt/b-catenin pathway [67]. Considering their expression patterns, we suggest that both miR-218 and miR-491 might have a role in *NAPRT* downregulation in the brain.

Most of the mentioned TFs, RBPs and miRNAs are also associated with cancer development. One particular pathway is the Wnt signaling pathway, mostly via the b-catenin pathway (also known as the canonical pathway), which has functions in tissue development and homeostasis, and is often altered in cancer (reviewed in [68]). Cellular processes such as proliferation, differentiation, adhesion and survival take place upon Wnt activation.

One of the cellular processes where the Wnt signaling pathway is determinant is the epithelial to mesenchymal transition (EMT), which is a key step not only in embryonic development but also in carcinogenesis. In the EMT, epithelial cells with strong cell–cell adhesion are converted in mesenchymal cells, which present more motile and invasive properties. After the discovery that *NAPRT* expression was lost in several EMT-subtypes of gastric tumors, Lee et al. suggested that *NAPRT* is involved in the Wnt pathway and plays a putative role in the stabilization of the β-catenin destruction complex [14]. In their study, a positive correlation between the expression of NAPRT and E-cadherin was described. In order to strengthen this relation, in our study, the global analysis of all targets showed that the CDH1 protein was targeted by three types of *NAPRT* expression regulators, namely by TCF3 (a TF), ESRP2 (an RBP) and the miR-92. These three potential NAPRT regulators were found to be significantly correlated with *NAPRT* expression. In the canonical Wnt signaling pathway, activation/repression of the target genes depends on the TCF/LEF family of TFs, which comprises TCF3, TCF7L2 and LEF1, which bind to b-catenin. TCF7L2 binding to *NAPRT* was retrieved from experimental data and TCF3 presented a positive correlation with *NAPRT* expression, further supporting *NAPRT* functions in this signaling pathway.

Our GO results also suggested a role for *NAPRT* in immune signaling, consistent with recent findings that the extracellular NAPRT protein (eNAPRT) triggered inflammatory responses in macrophages, mostly from the NF-kB pathway, and enhanced macrophage differentiation from circulating monocytes [16,17]. This is relevant, as it supports the new NAPRT roles independent of its enzymatic function on NAD production.

## 5. Conclusions

Our study suggests that *NAPRT* is involved in differentiation and developmental processes and showed that, beyond its application in cancer therapeutic strategies involving *NAMPT*, *NAPRT* may participate in the process of carcinogenesis and tumor progression. Particularly in neural development, our results are supported by the established association of *NAPRT* mutations with neurological/neurodevelopmental diseases, namely, attention-deficit/hyperactivity disorder and schizophrenia [69,70]. Studies in zebrafish also suggested that partial loss of function of the *NAPRT* gene results in abnormal brain development [70].

As such, our results indicate *NAPRT* potential regulators that should be experimentally studied. This particularly includes TFs TCF3/TCF7L2, the lesser known RBP ZCCHC17 and the miRNAs miR-218 and miR-491. The previously described link of NAPRT with the Wnt signaling [14] was emphasized by several of our analyses. Thus, given the role of this pathway in differentiation and developmental processes, NAPRT should also be further explored in this context.

## Figures and Tables

**Table 1:** Biological processes overrepresentation of the 80 potential *NAPRT* transcription factors. Only child terms relative to 10 or more genes and with a fold enrichment of over 3 are presented, by decreasing fold-enrichment order. Complete results are listed in Supplementary Table S4.

Transcrition Factors—BIOLOGICAL PROCESS
Immune response
Response to other organism
Regulation of epithelial cell proliferation
Positive regulation of immune system process
Negative regulation of cell population proliferation
Cellular response to growth factor stimulus
Regulation of immune response
Negative regulation of protein modification process

**Table 2:** Transcription factors and RNA binding proteins with a significant correlation with *NAPRT* expression.

	Gene Symbol	Protein Name	Spearman	*p* Value
Transcription factors	BCL3	BCL3 transcription coactivator	0.725	2.64 × 10^−11^
	CEBPB	CCAAT enhancer binding protein β	0.618	8.62 × 10^−8^
	JUN	Jun proto-oncogene, AP-1 transcription factor subunit	0.532	8.46 × 10^−6^
	MAFB	MAF bZIP transcription factor B	0.569	1.42 × 10^−6^
	PML	PML nuclear body scaffold	0.568	1.44 × 10^−6^
	RXRA	retinoid X receptor α	0.628	4.67 × 10^−8^
	STAT6	signal transducer and activator of transcription 6	0.573	1.14 × 10^−6^
	TCF3	transcription factor 3	0.543	5.23 × 10^−6^
	TMEM37	transmembrane protein 37	0.566	1.65 × 10^−6^
	YY1	YY1 transcription factor	0.522	1.33 × 10^−5^
	ZBTB7A	zinc finger and BTB domain containing 7A	0.590	4.52 × 10^−7^
RNA binding proteins	ESRP2	epithelial splicing regulatory protein 2	0.532	8.72 × 10^−6^
	HNRNPAB	heterogeneous nuclear ribonucleoprotein A/B	0.638	2.41 × 10^−8^
	HNRNPH1	heterogeneous nuclear ribonucleoprotein H1	0.503	3.15 × 10^−5^
	HNRNPL	heterogeneous nuclear ribonucleoprotein L	0.589	4.76 × 10^−7^
	PCBP1	poly(rC) binding protein 1	0.522	1.34 × 10^−5^
	PTBP1	polypyrimidine tract binding protein 1	0.608	1.63 × 10^−7^
	PTBP3	polypyrimidine tract binding protein 3	0.627	5.02 × 10^−8^
	SRSF2	serine and arginine rich splicing factor 2	0.621	7.30 × 10^−8^
	YBX1	Y-box binding protein 1	0.523	1.32 × 10^−5^
	ZCCHC17	zinc finger CCHC-type containing 17	−0.636	2.82 × 10^−8^

**Table 3:** Significant correlations between *NAPRT* and miRNA expression.

	Dataset		Spearman Correlation	*p*-Value
hsa-miR-92a-3p	GSE34608	Pulmonary tuberculosis and sarcoidosis	−0.797	1.3 × 10^−5^
	GSE38974	Chronic obstructive pulmonary disease	−0.626	4.1 × 10^−4^
	GSE42095	Differentiated embryonic stem cells	0.830	4.8 × 10^−7^
	GSE28544	Breast cancer	0.710	5.1 × 10^−5^
	GSE15076	Monocyte-derived dendritic cells	0.600	4.4 × 10^−2^
hsa-miR-218-5p	GSE38226	Liver fibrosis	−0.726	9.7 × 10^−5^

**Table 4:** Pathway overrepresentation of 626 targets of TFs and RBPs with a correlation with NAPRT expression and miRNA targets. Only the child terms relative to 10 or more genes and with a fold enrichment of over 3 are presented by decreasing fold enrichment order. Complete results are in Supplementary Table S13.

Target Genes—PATHWAYS
p53 pathway
Toll receptor signaling pathway
Apoptosis signaling pathway
Interleukin signaling pathway
Transcription regulation by bZIP transcription factor
Blood coagulation
CCKR signaling map
Ras Pathway
Gonadotropin-releasing hormone receptor pathway
Alzheimer disease-presenilin pathway
Inflammation mediated by chemokine and cytokine signaling pathway
TGF-β signaling pathway
Angiogenesis
Huntington disease

## Data Availability

Not applicable.

## Supplementary Materials

The following are available online at https://www.mdpi.com/article/10.3390/genes12122022/s1, Figure S1: Expression heatmaps for the experimentally validated miRNAs, obtained from DASHR (http://dashr2.lisanwanglab.org/, accessed on 14 December 2021). Table S1: Databases used for data collection on transcription factors, RNA binding proteins and microRNAs, Table S2: Full list of potential *NAPRT* transcription factors (total of 80), Table S3: Transcription factors with experimentally supporting data of *NAPRT* binding, Table S4: Gene ontology results for transcription factors, Table S5: Full list of potential *NAPRT* RNA binding proteins (total of 122), Table S6: RNA binding proteins with experimentally supporting data of *NAPRT* binding, Table S7: Gene ontology results for RNA binding proteins, Table S8: Full list of potential *NAPRT* microRNAs (total of 39), Table S9: microRNAs with experimentally supporting data of *NAPRT* binding, Table S10: Target genes of the TFs significantly correlated with *NAPRT* expression (total of 340), Table S11: Target genes of the RBPs significantly correlated with *NAPRT* expression (total of 149), Table S12: Target genes of the miRNAs experimentally validated (total of 158), Table S13: Gene ontology results of the target genes (total of 626) from the transcription factors and the RNA binding proteins that have a significant correlation with *NAPRT* expression and the microRNAs. Biological process (A) and molecular function (B) from the PANTHER slim annotations datasets, PANTHER pathways (C), by decreasing fold enrichment order. Only child terms are presented, Table S14: Top GO results for miR-92 (A) and miR-218 (B) target genes. The processes with a fold enrichment over 100 and relative to more than 10 genes are shown. Only child terms are presented.
