# Supplementary figures and images for: NAPRT Expression Regulation Mechanisms: Novel Functions Predicted by a Bioinformatics Approach
Source: Genes (Basel). 2021 Dec 20;12(12):2022. doi: 10.3390/genes12122022 (PMC8700865; doi:10.3390/genes12122022)

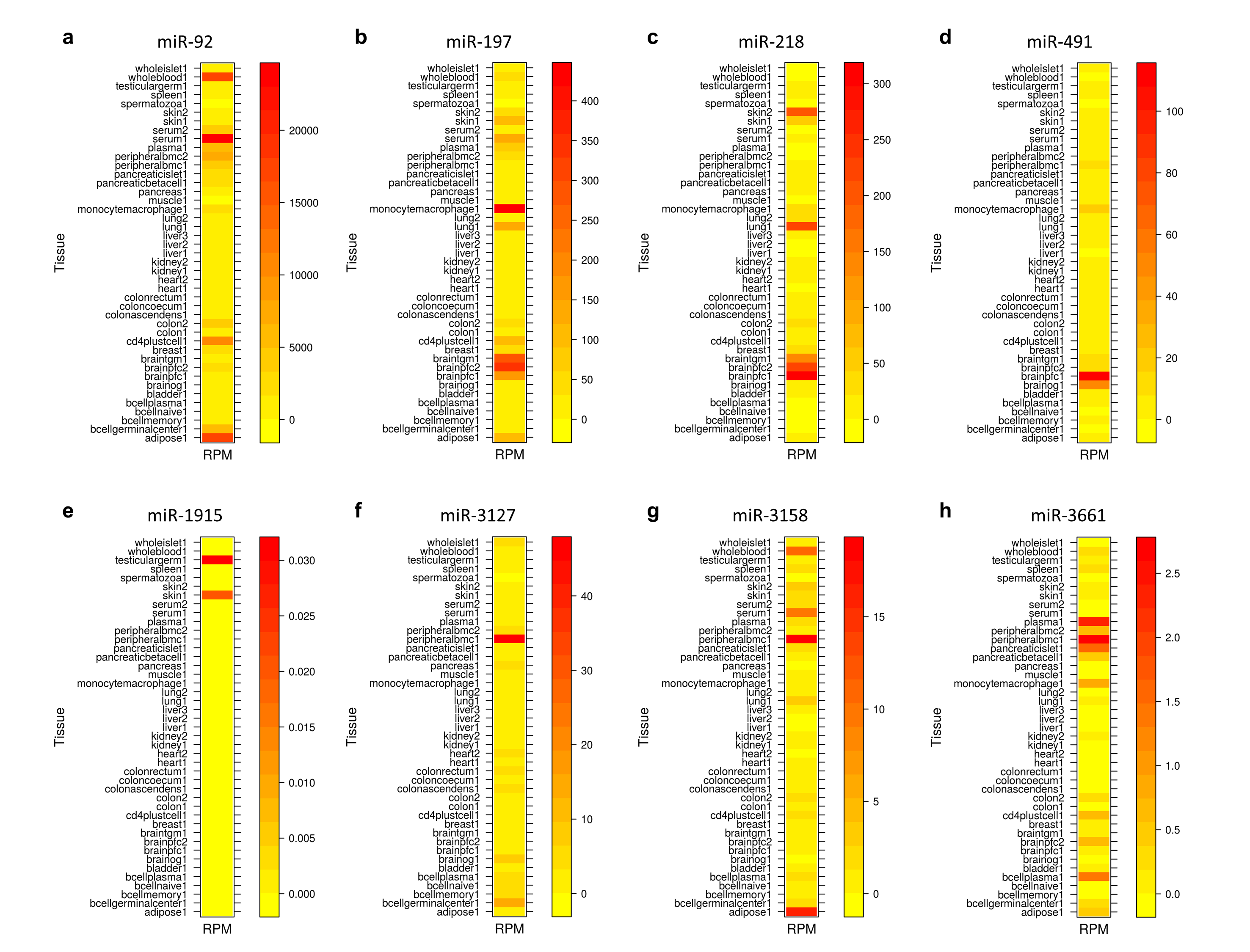

Supplement: Supplementary file 1 [file genes-12-02022-s001.zip › Figure S1.tif]
